# Draft Whole-Genome Sequences of Five Klebsiella pneumoniae Isolates from the Subantarctic Islands of New Zealand

**DOI:** 10.1128/MRA.01328-18

**Published:** 2018-11-21

**Authors:** Komkiew Pinpimai, Wendi D. Roe, Patrick J. Biggs, Keren E. Dittmer, Sarah A. Michael

**Affiliations:** a Pathobiology Group, School of Veterinary Science, Massey University, Palmerston North, New Zealand; b ^*m*^EpiLab, Infectious Disease Research Centre, School of Veterinary Science, Massey University, Palmerston North, New Zealand; c Wildbase, School of Veterinary Science, Massey University, Palmerston North, New Zealand; University of Arizona

## Abstract

Klebsiella pneumoniae is a Gram-negative bacterium that can be found in the environment, as well as on mucosal surfaces of humans and animals. Here, we report the genome sequence of five K. pneumoniae isolates from substrate samples and bird feces collected in the Subantarctic Islands of New Zealand.

## ANNOUNCEMENT

Klebsiella pneumoniae is an opportunistic pathogen causing nosocomial and community-acquired infections (1, 2). Over the past few decades, hypervirulent strains causing primary liver abscesses and septicemia have increasingly been documented (3, 4). Most hypervirulent strains have a hypermucoviscous phenotype (positive string test) and the *rmpA* and *rmpA2* genes (1, 3, 4). Hypervirulent strains have also been described in animals (5–7).

Hypervirulent K. pneumoniae infection has been reported as a cause of mortality events in New Zealand sea lion (NZSL) pups on Enderby Island, New Zealand, one of the Subantarctic Auckland Islands (7, 8). Annually, a large number of pups die from K. pneumoniae infection in the summer birthing season; however, reservoirs have not been investigated. Environmental substrates, birds that live on the island, and NZSL adults are possible reservoirs of this infection.

The use of animals and sample collection were completed under permit 39915-FAU from the New Zealand Department of Conservation and Massey University Animal Ethics Committee (approval 14/114). In this study, five K. pneumoniae isolates from cloacal swabs or voided feces from subantarctic skuas (*n* = 2) and a yellow-eyed penguin (*n* = 1) and substrate samples (water; *n* = 2) (using CHROMagar Orientation) were whole-genome sequenced. The NucleoSpin soil kit (Macherey-Nagel, GmbH & Co. KG, Germany) was used to extract genome-quality DNA from a single colony cultured on agar, which was sent to New Zealand Genomics Limited (Massey Genome Service, Massey University, Palmerston North, New Zealand). A fragment library was prepared using an Illumina TruSeq DNA library preparation kit v1 (Illumina, Inc., Scorsby, Victoria, Australia). Paired-end reads (2 × 250 bp) were obtained from a MiSeq instrument (Illumina, Inc., San Diego, CA). The five isolates from this study were *de novo* assembled using SPAdes v3.10 (in the “careful” mode) (9). The contigs of each isolate produced from SPAdes was annotated by Prokka v1.1.2, with default parameters (10). The sequence types (ST) and serotypes of bacterial isolates were determined using the Bacterial Isolate Genome Sequence Database (BIGSdb) servers (http://bigsdb.pasteur.fr/klebsiella/klebsiella.html). Virulence genes were identified by mapping the reads of each isolate, along with 1,000-bp flanks on either side of the virulence gene sequence, using Bowtie 2 in both the “–very-sensitive-local” and “–very-sensitive” modes for local and global read mapping, respectively. Genome sizes, numbers of contigs, ST, sources, serotypes, and virulence genes are summarized in Table 1.

**TABLE 1:** Descriptions of *K. pneumoniae* strains sequenced, their genomic characteristics, and associated virulence factors

Isolate	GenBank accession no.	SRA accession no.	Source, location	Serotype	ST	String test result	Length (bp)	No. of contigs	Fold coverage	GC content (%)	Virulence genes
E14_15_17Sa	QVNJ00000000	SRR7699496	Subantarctic skua,^*a*^ Enderby Island	Non-K1/1K2	2843	−	5,644,626	116	87	57.1	*wabG, uge, irp2, iucD, iutA, mrkD*
E14_15_42Sa	QVNI00000000	SRR7699497	Subantarctic skua,^*a*^ Enderby Island	K2	86	+	5,327,210	94	93	57.5	*rmpA, wabG, uge, iroN, irp2, ybtS, mrkD*
E14_15_53Ma	QVNH00000000	SRR7699498	Yellow-eyed penguin,^*a*^ Enderby Island	K2	86	+	5,351,349	91	89	57.5	*rmpA, wabG, uge, iroN, irp2, ybtS, mrkD*
E13_14_10sub	QVNG00000000	SRR7699499	Water, Enderby Island	K2	86	+	5,332,787	85	98	57.5	*rmpA, wabG, uge, iroN, irp2, ybtS, mrkD*
C14_15_17sub	QVNF00000000	SRR7699495	Water, Campbell Island	K2	86	+	5,323,133	99	94	57.5	*rmpA, wabG, uge, iroN, ybtS, mrkD*

a This animal was apparently healthy. *K. pneumoniae* was isolated from cloacal swabs or voided feces from nonclinical, live animals.

The virulence genes found in environmental and bird isolates were consistent with those found in other studies on clinical human isolates (11), suggesting that the isolates have pathogenic potential. This further suggests that the environment and birds may be possible reservoirs of this pathogen. The ST of the environmental isolates from this study was different from that in previous reports on other environmental isolates (12), suggesting genetic diversity of this bacterium in the environment (13).

These are the first whole-genome sequences of K. pneumoniae isolated from birds and environmental substrate samples from the Subantarctic Islands. The data from this study will provide information on genomic relationships between isolates from animals and environmental isolates in the Subantarctic and help to inform future research on the role of K. pneumoniae in harsh non-human-dominated island environments.

### Data availability.

The whole-genome shotgun sequences described here have been deposited in DDBJ/ENA/GenBank under the accession numbers listed in Table 1. Raw sequence reads have been deposited in the NCBI Sequence Read Archive under the accession numbers listed in Table 1.
